# Pre-peritoneal Plus Portal Infiltration Versus Bilateral Transversus Abdominis Plane Block in Laparoscopic Hernia Repair

**DOI:** 10.7759/cureus.47846

**Published:** 2023-10-28

**Authors:** Arthi Asokan, Arunkumar Muthalu, Jayashridevi Rajaraman, Deepu Thiyagarajan, Raghuraman M Sethuraman

**Affiliations:** 1 Anesthesiology, Sri Venkateshwaraa Medical College Hospital and Research Centre, Puducherry, IND; 2 Critical Care, Mahatma Gandhi Medical College and Research Institute, Puducherry, IND; 3 General Surgery, Sri Venkateshwaraa Medical College Hospital and Research Centre, Puducherry, IND; 4 Anesthesiology, Sree Balaji Medical College and Hospital, Bharath Institute of Higher Education and Research (BIHER), Chennai, IND

**Keywords:** laparoscopic hernia repair, inguinal hernia, transversus abdominis plane block, portal infiltration, pre-peritoneal infiltration

## Abstract

Background and objective

The role of the pre-peritoneal infiltration of local anesthetic (PILA) in laparoscopic hernia repair has been equivocal. Ultrasound-guided transversus abdominis plane (TAP) block has been extensively studied. However, studies comparing these two methods are very scarce. Hence, this study was undertaken to compare the efficacy of pre-peritoneal plus portal infiltration with TAP block in this population.

Materials and methods

This double-blinded randomized comparative study was conducted on a total of 32 patients by allotting 16 patients in each group. Group A patients were given pre-peritoneal plus portal infiltration of 15 mL of 0.5% ropivacaine for each technique by the operating surgeon, while Group B patients were administered bilateral TAP block with 0.5% ropivacaine, 15 mL on each side under ultrasound guidance by the anesthesiologist.

Results

The demographic variables and duration of surgery were comparable between the two groups. Also, the postoperative requirement of fentanyl between the two groups was insignificant. However, the duration of anesthesia was significantly longer in Group B attributing to the extra time taken for the administration of the TAP blocks.

Conclusion

Both ultrasound-guided TAP block and the PILA plus portal infiltration are effective techniques for pain relief after laparoscopic hernia repair. Either of these two techniques can be chosen depending on the availability of resources, expertise, etc.

## Introduction

Laparoscopic hernia repair has many advantages over open hernioplasty such as lesser postoperative pain, early ambulation, speedy recovery, and less incidence of chronic pain [1-3]. Nevertheless, there is still a need for rescue analgesics in the immediate postoperative period. The laparoscopic approach is becoming a more preferred technique by the surgeons and demanded by the patients as well. The two common approaches in laparoscopic hernia repair are (1) transabdominal pre-peritoneal (TAPP) and (2) total extra-peritoneal (TEP) hernia repair [1,2]. The TAPP technique is performed within the peritoneal cavity, breaching the peritoneum, while TEP is performed without breaching the peritoneal cavity [2]. Regardless of the type of surgery, pain after both TAPP and TEP repair was found to be of the same intensity and character, and it was located in the groin, of moderate to severe intensity, more on the first day that increased on coughing and mobilization. The cause of pain is considered to be related to abdominal muscle distension during a laparoscopic procedure, mesh fixation, and irritant effects of residual carbon dioxide (CO_2_) [3,4].

The pre-peritoneal infiltration of local anesthetic (PILA) was investigated about 25 years back and found to have no significant effect [5]. Subsequently, it has been investigated in several studies, and the results have been conflicting with some studies concluding a significant reduction in pain [6,7] while other studies did not observe much beneficial effects [8-11].

The transversus abdominis plane (TAP) block has become popular for providing pain relief in many procedures. It has been found to have beneficial effects in laparoscopic hernia repair [2,12]. However, to the best of our knowledge, only one study compared PILA with TAP block in this population so far [12], and no study has compared PILA plus trocar site infiltration to TAP. Hence, we conducted this study to evaluate the effects of ultrasound-guided bilateral TAP versus PILA plus trocar site infiltration in laparoscopic TEP hernia repair.

## Materials and methods

This double-blinded randomized control study was conducted on a total of 32 patients by allotting 16 patients in each group, after obtaining clearance from the Institutional Ethics Committee (SVMCH/IEC/2019-Nov/14). The trial was registered prospectively with the Clinical Trial Registry of India (CTRI/2019/12/022365). Group A patients received pre-peritoneal plus portal infiltration of 0.5% ropivacaine, while Group B patients received bilateral ultrasound-guided TAP block. The primary objective of this study was to compare the requirement of fentanyl during the initial 24 hours postoperatively. The secondary objectives were to find the incidence of seroma at the surgical site and postoperative complications if any.

All patients of 18-60 years of age of both sexes with American Society of Anesthesiologists (ASA) physical status I or II undergoing laparoscopic TEP hernia repair were included. Patients belonging to ASA III and beyond, patients with hypersensitivity to the study drugs, patients with significant cardiac and pulmonary disease, and pregnant or lactating females were excluded. After a complete pre-anesthetic check-up, written and informed written consent was obtained from all the eligible patients. Patients were kept nil per oral for eight hours prior to surgery. All patients were premedicated with tablet pantoprazole 40 mg, tablet alprazolam 0.5 mg at 10 PM before the day of surgery, and tablet metoclopramide 10 mg on the morning of surgery with sips of water. In the preoperative holding area, after recording the vital parameters, the patients were allocated into either Group A or B by randomization using the sealed envelope technique (blinded to the investigator). Patients were shifted to the operating room, and intravenous access with an 18 G cannula was secured. Standard monitoring ECG, noninvasive blood pressure cuff (NIBP), oxygen saturation (SpO_2_), and end-tidal carbon dioxide (EtCO_2_) were monitored and recorded. All patients were administered ondansetron 4 mg, glycopyrrolate 0.2 mg, and fentanyl 2 mcg/kg intravenously. After preoxygenation with 100% O_2_ for three minutes, induction was done with propofol 2mg/kg. An injection of vecuronium 0.1 mg/kg was given to facilitate endotracheal intubation. After securing the airway with an appropriate-size endotracheal tube, the maintenance of anesthesia was achieved with 66% N_2_O in 33% O_2_ + isoflurane (1.2%) + 0.01 mg/kg of vecuronium bromide as intermittent doses.

**Figure 1:**
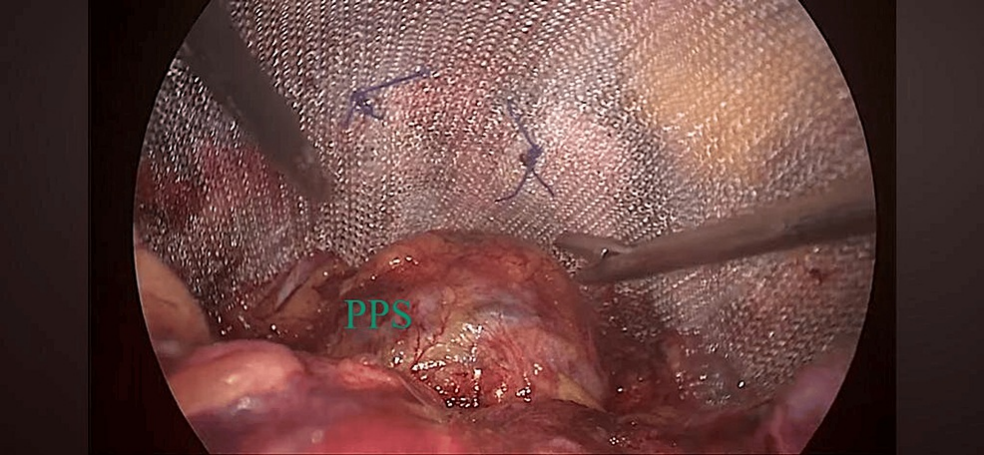
Pre-peritoneal space (PPS) where infiltration was given after securing the mesh

**Figure 2:**
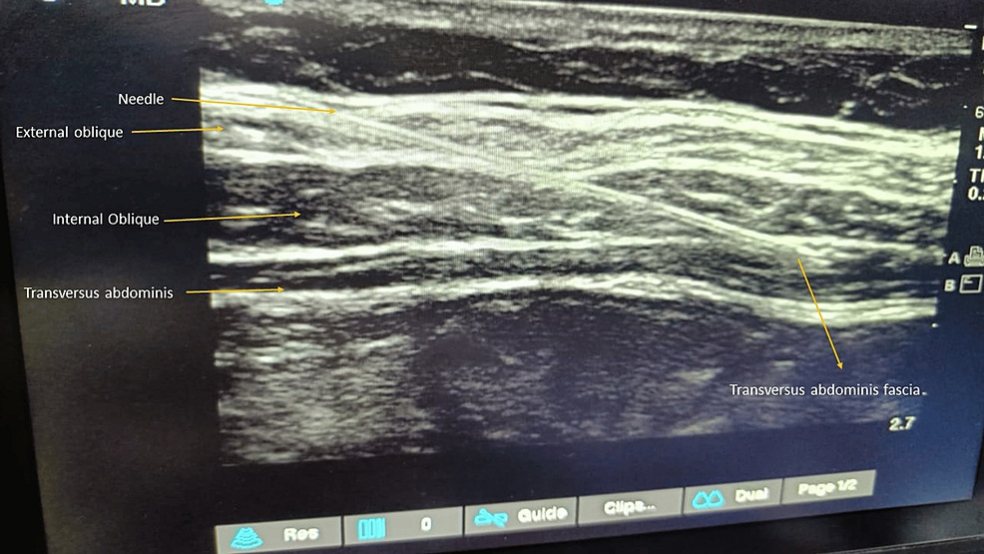
Ultrasound image of the TAP block TAP: transversus abdominis plane

All surgeries were performed by the same surgeon. After completing the surgical procedure, patients under Group A were given pre-peritoneal plus portal infiltration of 15 mL of 0.5% ropivacaine for each technique by the operating surgeon, 15 mL in the pre-peritoneal space (Figure 1) and 15 mL of drug in the periportal site. Patients under Group B were administered bilateral TAP block by the same anesthesiologist with 0.5% ropivacaine, 15 mL on each side under ultrasound guidance (Figure 2). All patients received an injection of diclofenac 75 mg intravenously (infusion over a period of 20 minutes) at the start of the closure of the wound. Patients were extubated after reversing the residual neuromuscular blockade with neostigmine bromide 0.05 mg/kg and glycopyrrolate 0.005 mg/kg.

In the postoperative room, all parameters were assessed by the anesthesiologist blinded to the study groups. The intensity of the pain was assessed using a visual analog scale (VAS) at 30 minutes, one hour, two hours, six hours, 12 hours, 18 hours, and 24 hours. The VAS score of ≥4 is taken as a cutoff for analgesic supplementation of an injection of fentanyl (1.5 mcg/kg) slowly intravenously. The time of demand in hours after surgery and the requirement of repeated doses of analgesia were recorded. Nausea, vomiting, and urinary retention were documented if these happened. The surgeon assessed the condition of the wound at a one- or two-week follow-up.

Sample size calculation

The sample size was calculated using a previous study [6], setting the power of the study at 80% and significance at 5%, and assigning the mean pain scores at one hour as 4 in the study group and 5 in the control group with a mean difference of 1, we arrived at a sample size of 16 in each group.

Statistical analysis

The data were analyzed by the Statistical Package for Social Sciences (SPSS) version 23 (IBM SPSS Statistics, Armonk, NY). Quantitative data such as age, weight, height, and pulse rate are expressed as mean ± standard deviation, while qualitative data such as gender and ASA status are expressed as a proportion. The number of rescue analgesics was analyzed using a t-test. P = 0.05 was taken as significant.

## Results

We assessed a total of 40 eligible patients for this study. Out of these, five patients were not willing to participate in the study, while three patients did not meet the inclusion criteria. Thus, a total of 32 patients were included and allotted in equal numbers to both groups.

The demographic variables such as age, sex, and weight and the ASA status of the patients were comparable between the two groups (Table 1). Although the number of patients with diabetes was more in Group B (10 versus eight), it was statistically insignificant (Table 1). Similarly, the number of patients with hypertension was also higher in Group B (11 versus eight) but again insignificant statistically (Table 1).

**Table 1:** Demographic characteristics ASA: American Society of Anesthesiologists

Variable		Comparison between the groups				p-value	Significance
		Group A		Group B			
Age (years)		42.8 ± 14.9		53.2 ± 15.1		0.0601	No
Weight (kg)		67.5 ± 6.7		65.9 ± 9.0		0.5835	No
Gender	Male	Number	Percentage	Number	Percentage		
		16	100%	13	81.2%	0.069	No
	Female	0	0%	3	18.8%		
ASA	I	5	31.2%	2	12.5%	0.200	No
	II	11	68.8%	14	87.5%		
Diabetes	No	8	50%	6	37.5%	0.476	No
	Yes	8	50%	10	62.5%		
Hypertension	No	8	50%	5	31.2%	0.280	No
	Yes	8	50%	11	68.8%		

Other parameters such as variations in heart rate, blood pressure, and oxygen saturation did not significantly vary between the groups (Table 2). The duration of surgery did not vary between the groups significantly with a mean of 66 versus 61.8 minutes (p = 0.4821) (Table 2). However, the duration of anesthesia was significantly higher in Group B with a mean of 70 versus 102.8 minutes (p < 0.0001) (Table 2). Two patients of Group A and four patients of Group B required fentanyl. Also, they required it only once in the first 24 hours. Among them, the mean time to rescue analgesia was 20 hours and nine minutes in Group A, while it was 20 hours and 27 minutes in Group B (p = 0.27). The postoperative requirement of fentanyl and VAS between the two groups was insignificant (Table 3).

**Table 2:** Comparison of physical variables between the groups SD, standard deviation; SpO_2_, oxygen saturation

Variable	Comparison between the groups				p-value	Significance
	Group A		Group B			
	Mean	SD	Mean	SD		
Duration of anesthesia (minutes)	70	18.3	102.8	15.1	<0.0001	Yes
Duration of surgery (minutes)	66	4.5	61.8	16.7	0.4821	No
Heart rate	84.9	6.3	88.4	6.9	0.1520	No
Systolic blood pressure	130.8	8.7	132	8.9	0.7209	No
Diastolic blood pressure	82.6	6.0	80.7	8.4	0.4751	No
SPO_2_	98.7	0.5	98.6	0.6	0.5319	No

**Table 3:** Comparison of fentanyl requirement and VAS between the groups VAS: visual analog scale

Variable	Comparison between the groups				p-value	Significance
	Group A		Group B			
	Number	%	Number	%		
Fentanyl required	2	12.5	4	25.0	0.365	No
Fentanyl not required	14	87.5	12	75.0		
VAS at six hours	1.2	0.7	1.1	0.3	0.5177	No
VAS at 12 hours	1.6	0.6	1.8	0.8	0.3462	No
VAS at 18 hours	1.8	0.7	1.8	0.8	1.000	No
VAS at 24 hours	2.5	1.2	2.4	1.2	0.6635	No

## Discussion

Our study observed that the PILA plus portal infiltration and the TAP block provided similar pain relief in laparoscopic TEP hernia repair. To the best of our knowledge, no study has compared these two techniques in this population.

The efficacy of PILA has been inconsistent in laparoscopic hernia repair. While some studies were favorable [6,7], a few studies did not note much beneficial effects [8-11]. On the other hand, ultrasound-guided TAP block has gained popularity and has been accepted worldwide for pain relief in this population in the last decade. Many studies found that TAP block was effective in laparoscopic hernia repair [2,12-15].

However, studies comparing these two established techniques (PILA and TAP block) are very scarce. Indeed, we could find only one such study [12]. Hence, we designed this study to compare these two techniques. Notably, in contrast to that study [12], we added the portal site infiltration to PILA as it is the routine practice for us if no regional block such as TAP and epidural analgesia was provided. Sakamoto et al. [12] observed a significant reduction in postoperative opioid consumption in the TAP group. However, we found that it was similar between the two groups. We believe that it could be due to the additional portal site infiltration we adopted in this study.

Arora et al. compared the TAP block to portal infiltration (both were performed before surgery) and found that the TAP block resulted in a significant reduction of intraoperative and postoperative opioid requirement [2]. Beyls et al. observed that the TAP block could reduce significantly the “postoperative analgesic consumption before discharge” when compared to the “standard care” comprising acetaminophen and nonsteroidal anti-inflammatory drugs [13]. Takebayashi et al. concluded that either the TAP block or the rectus sheath block reduced the postoperative pain significantly, besides hastening the recovery when compared to the control group [14]. TAP block reduced the pain scores and fentanyl requirement significantly when compared to the control group in TEP hernia repair as per a study published in 2012 [15].

Laparoscopic-assisted TAP block provided by the surgeon was another method adopted in a study published recently that found TAP block resulted in a significant reduction in pain scores and opioid requirement and improved patient satisfaction when compared to the portal site infiltration [16]. We believe that this technique would reduce the time of performance of the block. Importantly, in our study, the duration of anesthesia was significantly longer in the TAP block group because of the time taken for the ultrasound-guided TAP block.

Our study has a few limitations. First, we did not have a control group. This is because of the difficulty in getting ethical clearance if a control group is added. Also, we were worried about getting an adequate number of patients as this study was designed with conditions such as a single surgeon to operate all the cases and a single anesthesiologist performing all the TAP blocks for standardization. This is also the reason for the small sample size we chose for this study. Second, we could have tested only the efficacy of PILA without adding the portal infiltration.

## Conclusions

We conclude that ultrasound-guided TAP block and the PILA plus portal infiltration are effective techniques for pain relief after laparoscopic hernia repair. PILA plus portal infiltration can be considered as an alternative if equipment, expertise, etc. are constraints for ultrasound-guided TAP block. Future studies comparing PILA to laparoscopic-assisted TAP block may help us to understand the efficacy of these techniques well.
